# Related Factors for Impaired Fasting Glucose in Korean Adults: A Population Based Study

**DOI:** 10.1186/s12889-021-12276-5

**Published:** 2021-12-11

**Authors:** Hyunjin Lee, Bohyun Kim, Youngshin Song

**Affiliations:** 1 College of Nursing, Eulji University, 712, Dongil-ro, Uijeongbu-si, Gyeonggi-do Republic of Korea; 2 Department of Nursing, Hallym Polytechnic University, 48, Janghak-gil, Dong-myeon Chuncheon-si, Gangwon-do 24210 Republic of Korea; 3 College of Nursing, Chungnam National University, Jung-gu, Munhwa-ro 266, Daejeon Daejeon, 35015 Republic of Korea

**Keywords:** Impaired fasting glucose, Diabetes mellitus, Health behavior, Health Literacy

## Abstract

**Background:**

Individuals with impaired fasting glucose (IFG) who have poor health behaviors are at a greater risk for various health outcomes. This study compared the health behaviors and health literacy between individuals with non-IFG and IFG; factors that were associated with IFG were identified by sex.

**Methods:**

This study was an observational study with a cross-sectional design based on data from the Korea National Health and Nutrition Examination Survey (KNHANES) that used a stratified, multi-stage, cluster-sampling design to obtain a nationally representative sample. This study analyzed the KNHANES Health Examination Survey and Health Behavior Survey from 2016 to 2018 (N=9919). Multiple logistic regression analysis was employed to compute the odds ratios of health behaviors and health literacy to identify the risk factors for IFG.

**Results:**

The prevalence of IFG among the total was 29.0% (weighted n=2826, 95% CI 27.8–30.2). In the IFG group, 63.6% were male and 36.4% were female (X^2^=320.57, *p*<.001). In multiple logistic regression by sex, the factors associated with IFG in male were as follows: age (50s; OR=2.36, 95% CI 1.79–3.13), high BMI (OR=2.27, 95% CI 1.78–2.90), frequent drinking (OR=1.83, 95% CI 1.23–2.72), and using nutrition fact labels (OR=1.35, 95% CI 1.05–1.75). Low economic status (OR=4.18, 95% CI 1.57–11.15) and high BMI (OR=2.35, 95% CI 1.29–4.28) were the affecting factors in female. On the other hand, employment status, perceived stress, and job type were not related to IFG in both male and female.

**Conclusions:**

Strategies should be targeted to improve health behaviors and health literacy for those in their 40s and 60s, male in shift work, those who frequently dine out, overweight male, female with low economic statuses, and frequent drinkers. Moreover, healthcare providers should understand the barriers to health behaviors and literacy to effectively deliver healthcare service.

## Background

Impaired fasting glucose (IFG) is known to progress to type 2 diabetes. It refers to a state in which an individual’s fasting blood glucose ranges from 100 to 125 mg/dl, just below the level for diabetes, which starts at 126mg/dl [1]. IFG has an extremely high probability of progressing to type 2 diabetes [2]. In an earlier longitudinal study, individuals with IFG were found to be more likely to transition to diabetes [3], and the incidence of diabetes is 10 times higher than those with normal blood glucose levels [2]. Although healthcare providers emphasize the importance of changing one’s lifestyle to prevent diabetes [4], individuals with IFG are nevertheless at risk of engaging in unhealthy eating habits, smoking, and the consumption of alcohol [5]. In particular, male in Korea engage in unhealthy behaviors such as drinking and smoking [6]. The Diabetes Risk Assessment Tool in the ADA (American Diabetes Association) guidelines includes a sex-specific category, and being male is recognized as a risk factor for diabetes [7].

Individuals with IFG should obtain and comprehend a wide range of health information to understand their situation and practice health behaviors [8]. In this regard, health literacy –the ability to understand basic health-related information and obtain and utilize services – is significantly correlated with patients’ abilities to practice health behaviors and maintain target blood glucose levels [9]. Previous studies have shown that individuals with lower levels of health literacy experience poor health conditions, poor management of health, and high medical expenses [10, 11]. However, these results have not been sufficiently consistent to provide evidence in terms of sex differences, issues on health literacy measurement methods, or health literacy’s impacts on health outcomes [12].

This is because health literacy capacities may be mediated by individuals’ education and socio-cultural behavioral background and because measurement tools for health literacy also vary [12]. In particular, the patriarchal family culture in Korea reduces the opportunity for male to access food information, that is, to search for information, compared to female. Middle-aged Korean male are less interested in purchasing groceries [13], and are still dependent on married middle-aged female for meal preparation [13, 14]. Therefore, there is inevitably a difference in health literacy related to food information according to sex. In a previous study, health literacy, which measured the ability to use food nutrition labels, was found to differ by sex, educational level, and age but was not associated with health behavior [15]. As such, there is a gap in the knowledge from studies in which health literacy and health behaviors affect IFG.

To address this gap, this study compares how the general characteristics of IFG and non-IFG differ and how health behaviors and health literacy differ using population data. Moreover, considering Korea's sex meal preparation culture, factors affecting IFG were analyzed by sex.

## Methods

### Study Design

The present study is an observational study with a cross-sectional study based on data from the Korea National Health and Nutrition Examination Survey (KNHANES) that was conducted to compare the differences of demographics, health behaviors, and health literacy between individuals with IFG and non-IFG. Furthermore, considering the cultural characteristics of Korea, IFG related factors according to sex were identified.

### Data Source and Participants

This investigation utilizes the Korean Health and Nutrition Examination Survey (KNHANES) data, which is a survey conducted by the Korean Ministry of Health and Welfare and the Korea Centers for Diseases Control and Prevention (KCDC). The KNHANES evaluates the health and nutrition status of Koreans, monitors trends in health risk factors and major chronic diseases, and provides data for the development and evaluation of Koreans health policies and programs. This survey used a stratified, multistage probability sampling design to select housing units. This included stratifying by region in the first stage and layering by sex and age in the second stage. To represent the entire Korean adult population and account for the complex sampling procedure, sampling weights were used. The weighted sample for the KNHANES reflected the sampling fraction and nonresponse bias adjustments.

In total, data from 24,269 people who participated in the KNHANES VII (2016–2018) were analyzed to determine whether the inclusion criteria were met. Data that met the exclusion criteria, such as those under 29 or over 65 years and diagnosed with type I or II diabetes, were then removed (excluded data n=14,350). The KNHANES survey comprised the following: The Health Interview Survey, Health Behavior Survey, Nutrition Survey, and Health Examination Survey, and this study’s variables were collected from these four surveys.

### Assessment of Measurements

The KNHANES VII (2016–2018) was used to obtain information on demographic characteristics, hematologic examinations, health behaviors, and health literacy (i.e., recognition of nutrition fact labels and utilization of the nutrition fact labels).

#### Demographic characteristics

Sex, age, economic status (low, middle, high), education level (≤ middle school, ≥high school), employed status (yes or no), duty type (day, shift), perceived health status (good, bad), body mass index (< 23 kg/m^2^ or ≥ 23 kg/m^2^), and perceived stress (never feel, feel a little, feel much) were used to assess socioeconomic status.

Economic status was based on the average monthly household income. Household income was partially adjusted by sex and age based on monthly equivalent income (= monthly household income/ number of families). Education level was categorized based on graduation status, such that completion, withdrawal, enrollment, and leave of absence were categorized with the preceding academic background.

Citizens in the Republic of Korea receive mandatory education through middle school in accordance with Article 31 of the Constitution. Therefore, the education status was categorized based on whether each participant was a middle school graduate or had received more than compulsory education.

For employed status, participants were asked “Have you worked for more than an hour in the last week for income? Or have you worked for your family for more than 18 hours unpaid? If you were originally working but are on a temporary leave of absence, this is considered work.” For duty type, people who answered “yes” in response to “Do you work from 6 am to 6 pm?” were classified as day type, and people who answered “yes” in response to “Or do you work during a different time period?” were classified as shift type.

The question regarding perceived health status asked, “What do you usually think about your health?” and the result was categorized into good or bad. Body mass index (BMI) was calculated using measured height and obesity. In this study, participants with a BMI of less than 18.5 kg/m^2^ were categorized as underweight, those with a BMI of 18.5–22.9 kg/m^2^ were categorized as normal weight, and those with a BMI of at least 23.0 kg/m^2^ were categorized as overweight [16]. Participants were considered as not overweight or overweight based on whether their BMI was < 23 kg/m^2^ or ≥ 23 kg/m^2^, respectively.

#### Health behaviors

Dining out was classified as less than once a month, less than twice a week, 3 to 4 times a week, or every day. Participants’ alcohol consumption during the recent year was categorized as not at all, less than once a week, and more than two times a week. For smoking, “Smoking every day” and “Smoking occasionally” were classified as “yes,” and “I smoked in the past, but not now” was classified as “no.”

The American Diabetes Association (ADA) [17] recommends that people with prediabetes and IFG walk for at least 150 minutes a week. Weekly walking time was categorized as less than 150 minutes or more than 150 minutes a week. Weekly aerobic activity equivalent to medium-intensity physical activity for over 2 hours and 30 minutes or high-intensity physical activity for over 1 hour and 15 minutes or a mixture of medium- and high-intensity physical activity (high intensity for one minute or medium intensity for two minutes) was classified as “yes” or “no.”

Total sitting time per day was calculated based on the usual sitting time during the week, and was categorized into sitting less than four hours a day or sitting for more than four hours a day.

**Health literacy.** Health literacy was measured by two questions. Participants responded to questions regarding the recognition of nutrition fact labels and utilization of nutrition fact labels with “yes” or “no.” The outcome variable, “the presence of IFG” was assessed through responses to questions regarding whether participants have fasted for 8 hours without diabetes and have a fasting glucose level greater than 100 mg/dl but less than 126 mg/dl [1].

### Statistical Analysis

To increase the sensitivity of this study and reduce hidden biases such as sampling bias, complex samples analysis procedures were implemented in consideration of sampling weights, stratification variables, and cluster including two or more survey districts by city/state/house type variables using the IBM SPSS 25.0 program. Through the process of applying these weights, the sampling bias was reduced. Missing data were statistically excluded. First, data from the selected sample were analyzed for individuals with non-IFG and IFG. Chi-square tests were conducted to compare the percentage of all variables for individuals with non-IFG and IFG. For the logistic regression analysis of the main outcomes, Shapiro-Wilk test was performed to test for normality, and the normality assumption was satisfied with *p*-value > .05. To get the evidence for collinearity among the independent variables, whether Variance Inflation Factor (VIF) was is greater than 10. As results, there were not found s evidence for collinearity. That is, there were no variables greater than 10. The chi-square statistic was computed comparing the observed frequencies with those expected under the linear model using the Hosmer-Lemeshow tests. A non-significant chi-square found in this study (x^2^= 10.40, *p* > .05), indicating that the data fit the model well.

In the next step, the logistic regression model was conducted using variables with significant results in the univariate analysis to investigate the association between variables that were statistically significant at less than 0.05. Moreover, multivariate logistic regression was stratified into female and male.

## Results

### Demographic Characteristics and IFG

The demographic characteristics of the two groups are shown in Table 1.

**Table 1:** Demographic characteristics of participants (N=9919)

Variables	Categories	Non-IFG (n=7093) Weighted n (%)	IFG (n=2826) Weighted n (%)	X^2^	*p*
Sex	Female	4492 (56.1)	1233 (36.4)	320.57	<.001
	Male	2601 (43.9)	1593 (63.6)		
Age, years	30-39	2218 (33.9)	476 (18.1)	304.70	<.001
	40-49	2170 (31.7)	866 (32.8)		
	50-59	1914 (25.9)	980 (35.3)		
	60-64	791 (8.6)	504 (13.8)		
Economic status	Low	513 (6.8)	277 (9.3)	18.54	.002
	Middle	3931 (56.4)	1567 (55.4)		
	High	2635 (36.8)	978 (35.2)		
Education level	≤ Middle school	1013 (13.3)	597 (19.5)	56.07	<.001
	≥ High school	5767 (86.7)	2089 (80.5)		
Employed status	Yes	4845 (73.7)	2071 (79.2)	33.25	<.001
	No	1936 (26.3)	615 (20.8)		
Duty type	Day type	4479 (85.0)	1944 (88.0)	12.03	.002
	Shift type	790 (15.0)	276 (12.0)		
Perceived health status	Good	5869 (86.3)	2300 (86.0)	0.19	.685
	Bad	934 (13.7)	395 (14.0)		
BMI	< 23 kg/m^2^	3493 (48.6)	688 (23.9)	529.70	<.001
	≥23 kg/m^2^	3518 (51.4)	2123 (76.1)		
Perceived stress	Never feel	819 (10.8)	378 (13.2)	14.04	.002
	Feel a little	4242 (60.2)	1679 (60.4)		
	Feel much	1985 (29.0)	735 (26.4)		

BMI: Body Mass Index, IFG: Impaired Fasting Glucose

Among the 9919 participants, 7093 (71.0 %; 95% CI:69.8–72.2) were in the normal fasting glucose group and 2826 (29.0 %; 95% CI:27.8–30.2) were in the IFG group.

The mean age of the participants was 45.3 ± 0.2 years old. Of the total, 36.8% of participants were male, and 30.6% were 40–49 years old. Additionally, 80.5% of the participants had at least a high school education in the IFG group. The mean body mass index (BMI) of the participants was 23.8 ± 0.04 (kg/m^2^). The presence of IFG varied significantly by employed status (*p* < .001), duty type (*p* = .002), body mass index (*p* < .001), and perceived stress (*p* = .002).

### Health Behaviors and IFG

In the IFG group, 2296 (96.6%) dined out more than twice a week, while 6014 (97.6%) of the non-IFG group dined out twice a week or more. Regarding drinking behaviors, 36.4% of the IFG group drank alcohol, while 23.0% of the non-IFG frequently drank alcohol. There were also significant differences between the two groups in their current smoking status (*p* = .009). In the IFG group, 767 (51.7%) did not currently smoke, and in the non-IFG group, 1032 (53.2%) were currently smokers. For other health behaviors, the mean walking time per week of the non-IFG group was 237.9 ± 4.7 minutes, and the total time sitting per day was 495.9 ± 5. minutes. In IFG group, the mean walking time per week was 233.6 ± 5.7 minutes, and the total time sitting per day was 476.0 ± 5.1 minutes. Walking time per week (*p* = .451), practice of aerobic activity (*p* = .416), and total time sitting per day (*p* = .905) were not significantly different between the groups (Table 2).

**Table 2:** Health behaviors of participants (N=9919)

Variables	Categories	Non-IFG (n=7093) Weighted n (%)	IFG (n=2826) Weighted n (%)	X^2^	*p*
Dining out	Less than once a month	179 (2.4)	91 (3.4)	6.34	.016
	More than twice a week	6014 (97.6)	2296 (96.6)		
Drinking in the past year	Not at all	1069 (16.2)	337 (12.0)	131.45	<.001
	Less than 1 time/week	3998 (60.8)	1378 (51.6)		
	More than 2 times/week	1513 (23.0)	912 (36.4)		
Current smoking status	Yes	1302 (53.2)	709 (48.3)	8.93	.009
	No	1194 (46.8)	767 (51.7)		
Walking time per week	<150 minutes	3792 (54.1)	1555 (55.1)	0.75	.451
	≥150 minutes	3301 (45.9)	1271 (44.9)		
Practice of aerobic activity	Yes	3060 (46.2)	1181 (45.2)	0.81	.416
	No	3716 (53.8)	1508 (54.8)		
Total time sitting per day	< 4 hours	1093 (15.6)	440 (15.7)	0.01	.905
	≥ 4 hours	6000 (84.4)	2386 (84.3)		

IFG: Impaired Fasting Glucose

### Health Literacy and IFG

Significant differences were observed between the two groups in health literacy such as in the recognition and utilization of nutrition fact labels. The percentages of people who recognized nutrition fact labels were 87.4% in the non-IFG group and 81.8% in the IFG group, which shows a significant difference (*p* < .001). Regarding utilization of nutrition facts labels, 2,180 (38.9%) such participants were in the non-IFG group and 594 (30.5%) were in the IFG group (*p* < .001) (Table 3).

**Table 3:** Health literacy of participants (N=9919)

Variables	Categories	Non-IFG (n=7093) Weighted n (%)	IFG (n=2826) Weighted n (%)	X^2^	*p*
Recognition of nutrition fact labels	Yes	5427 (87.4)	1945 (81.8)	42.20	<.001
	No	766 (12.6)	442 (18.2)		
Utilization of nutrition facts labels	Yes	2180 (38.9)	594 (30.5)	45.30	<.001
	No	3247 (61.1)	1350 (69.5)		

IFG: Impaired Fasting Glucose

### Factors Associated with IFG

Table 4 presents the OR and 95% CI for predictors of IFG.

**Table 4:** Odds ratios for IFG (N=9919)

Variables (references)	Categories	Univariate analysis		Female						Male					
				Model I		Model II		Model III		Model I		Model II		Model III	
		OR	95% CI	OR	95% CI	OR	95% CI	OR	95% CI	OR	95% CI	OR	95% CI	OR	95% CI
Sex (female)	Mamle	1.66*	1.25 - 2.20												
Age (30 yr)	40 yr	1.77*	1.38 – 2.26	1.90*	1.44-2.52	2.03	0.96-4.26	1.98	0.92-4.25	2.19*	1.77-2.71	1.74*	1.36-2.23	1.72*	1.34-2.20
	50 yr	2.41*	1.85 – 3.13	2.25*	1.67-3.03	2.02	0.85-4.82	2.20	0.86-5.60	2.77*	2.25-3.40	2.41*	1.83-3.17	2.36*	1.79-3.13
	60 yr	2.12*	1.48 – 3.03	2.83*	1.94-4.13	2.56	0.33-19.57	5.31	0.65-43.07	2.92*	2.22-3.84	2.08*	1.47-2.96	2.04*	1.42-2.93
Economic status (high)	Low	1.28	0.85 – 1.93	1.37	0.99-1.89	4.15*	1.53-1.24	4.18*	1.57-11.15	1.28	0.85-1.92	1.13	0.68-1.86	1.14	0.69-1.90
	Middle	1.08	0.89 – 1.31	1.10	0.90-1.34	2.92*	1.24-6.84	2.78*	1.19-6.49	1.01	0.85-1.19	1.04	0.85-1.28	1.03	0.83-1.27
Education level (≥ high school)	≤ Middle school	1.26	0.94 – 1.69	1.05	0.80-1.37	1.17	0.48-2.85	1.20	0.49-2.97	1.23	0.94-1.62	1.29	0.94-1.76	1.29	0.94-1.77
Employed status (No)	Yes	1.11	0.79 – 1.72	0.95	0.70-1.29	1.14	0.51-2.52	1.18	0.53-2.61	1.12	0.73-1.71	1.18	0.72-1.92	1.15	0.71-1.88
Duty type (shift type)	Day duty type	0.68*	0.53 – 0.89	0.89	0.67-1.19	0.61	0.33-1.12	0.61	0.33-1.13	0.73*	0.58-0.93	0.67*	0.51-0.89	0.68*	0.51-0.90
BMI (< 23 kg/m^2^)	≥ 23 kg/m^2^	2.30*	1.86 – 2.83	2.85*	2.39-3.41	2.44*	1.33-4.47	2.35*	1.29-4.28	2.37*	1.94-2.89	2.25*	1.77-2.88	2.27*	1.78-2.90
Perceived stress (never feel)	Feel a little	0.92	0.68 – 1.25	0.95	0.72-1.26	0.80	0.26-2.45	0.808	0.25-2.55	0.90	0.70-1.16	0.9	0.66-1.24	0.9	0.66-1.24
	Feel a lot	0.92	0.62 – 1.28	0.71*	0.53-0.96	0.41	0.13-1.33	0.41	0.12-1.39	0.94	0.71-1.24	0.95	0.67-1.34	0.95	0.67-1.35
Dining out (less than once a month)	More than 2 times/week	0.52	0.24 - 1.12			0.78	0.16-3.67	0.73	0.17-3.17			0.42*	0.20-0.87	0.41*	0.19-0.85
Drinking in the past year (not at all)	Less than 1 time/week	1.76*	1.23 – 2.53			1.23	0.51-2.96	1.22	0.51-2.91			1.14	0.77-1.70	1.13	0.76-1.68
	More than 2 times/week	1.83*	1.22 – 2.74			0.93	0.33-2.65	0.97	0.34-2.76			1.85*	1.25-2.76	1.83*	1.23-2.72
Current smoking status (yes)	No	0.86	0.72 – 1.03			1.6	0.86-2.95	1.67	0.90-3.09			0.82	0.67-1.01	0.81*	0.66-0.99
Recognition of nutrition fact labels (yes)	No	0.90	0.68 – 1.19					1.57	0.53-4.59					0.86	0.65-1.15
Using nutrition facts labels (yes)	No	1.22	0.97 – 1.52					0.64	0.35-1.18					1.35*	1.05-1.75

BMI: Body Mass Index; OR: Odds Ratio; 95% CI: 95% Confidence Interval; *=*p*<.05

In univariate analysis, sex, age, duty type, BMI, and drinking in the past year were found to be factors related to IFG. Male were 1.66 times more likely to have IFG than female. Those in their 50s were 2.41 times more likely to have IFG than were those in their 30s.

In multivariate analysis by sex, the predictors of IFG in female were low (OR = 4.18, 95% CI 1.57–11.15) and middle (OR = 2.78, 95% CI 1.19–6.49) economic status and having an obese-level BMI (OR = 2.35, 95% CI 1.29–4.28). While age (40s OR = 1.72, 95% CI 1.34–2.20; 50s OR 2.36, 95% CI 1.79–3.13; 60s OR = 2.04, 95% CI 1.42–2.93), frequent dining out (OR = 0.41, 95% CI 0.19–0.85), frequent drinking (OR = 1.83, 1.23–2.72), and not using nutrition fact labels (OR = 1.35, 95% CI 1.05–1.75) were associated with IFG in male.

## Discussion

This study found that the prevalence of IFG was 29% and that predictors of IFG differed by sex. The prevalence of IFG 29% was lower than in Taiwan 35.8% [17], China 33.3% [17], and Western countries 29.2%–37.4% [18], but it has increased by 5% compared to 15 years ago [19]. This is consistent with the continued increase in diabetes prevalence from 11.1% in 2012 to 15.9% in 2018 [20]. Although this prevalence is lower than in other countries, personal efforts and political attention is important because it has gradually risen from 23% in 2006.

The predictors of IFG were similar to those found in previous studies. As is already known, being male, older, and obese, as well as engaging in poor health behaviors such as drinking and smoking, were also associated with IFG in this study [18]. Moreover, health literacy was significantly different between the non-IFG group and IFG group, but univariate analysis did not show health literacy as a predictor. The cause can be found from a previous report showing that male with IFG have a lower health literacy and poorer health behaviors than do female [19]. Therefore, the factors affecting IFG were analyzed by dividing male and female in this study, and meaningful results were obtained.

In male, drinking was a predictor of IFG. Many countries report that male drink more frequently and drink more than female [20], and Korean male also have higher drinking levels than Korean female [21]. Studies have reported that there was a strong association between male with IFG and risk factors for drinking, smoking, and obesity [22]; thus, active management may be needed. More than 79% of participants with IFG were employed. Korean male office workers have various health problems such as diabetes mellitus due to a lack of exercise, poor eating habits, and drinking and smoking [23, 24]. Although the legal working hours per week in South Korea were shortened with the introduction of the 5-day workweek system in 2004, South Korea continues to have the longest work hours in the world [25]. A substantial number of Korean office workers experience a lack of exercise due to their overtime work [23, 25] and they experience many drinking problems due to the get-together culture and public drinking [26]. Health professionals should focus on education and counseling on risk factors for IFG in male workers, emphasize the importance of healthy lifestyles. However, our results did not show a significant association between employment and IFG. In previous studies, the drinking behaviors of Korean male based on employment status were different [26]. Results of this study suggest that it is necessary to classify occupational groups and analyze them. In addition, with regard to smoking, our findings showed that male with IFG had a lower smoking rate than male without IFG. However, since smoking is reported as a risk factor for diabetes [22, 27], an in-depth study on this is needed in the future.

The results of this study indicate that the proportion of individuals with IFG who were actually using food and nutrition information was lower than that of individuals with non-IFG. Male with IFG had lower health literacy than did female with IFG. A number of European countries have reported that low education, old age, and financial scarcity are commonly associated with low health literacy [28, 29]. However, information on the relationship between sex and health literacy is still lacking, and the results of health literacy according to sex suggest otherwise [30, 31]. Individuals with IFG need health and food information they can understand and use in order to maintain target blood glucose levels and properly manage their health [32]. However, Koreans have a low understanding of food information and a low level of practice in using information for food selection [30]. In particular, middle-aged Korean male are less interested in food purchases [13], and married middle-aged female still take on the main responsibility for preparing meals at home and as food purchasers [13, 14]. Even in other Western countries, female still have more household responsibilities [33] and are much more likely to cook [34]. Middle-aged adult male in Korea depend on female for meal preparation, and it is expected that this patriarchal family culture will show differences in food consumption behavior, which is expected to affect health literacy. However, further research on health literacy is needed in the future.

Fasting blood glucose levels and obesity are affected by balanced caloric intake rather than daily caloric intake [35]. In addition, the outcome of body mass index (BMI) control through dietary intake and physical activity affects IFG [5]; thus, it is important to choose a balanced diet. Western countries are using strategies to improve individuals’ health literacy for managing their blood glucose and utilizing nutritional information on various foods [36, 37]. However, in South Korea, there is still a lack of understanding of food information and systems that can be properly utilized in everyday life [38]. In several countries where there is a high illiteracy rate, low health literacy can be understood as an inability to read or understand essential health-related data, and thus, to manage health [19]. However, the illiteracy rate is low in South Korea because of the high level of education; more than 80% of the participants in this study had an educational background of 10th grade or higher. The low level of health literacy compared to the high level of literacy can be interpreted as a lack of health education and public relations in terms of health. Low health literacy may be affected by a lack of personal competencies to use information as well as health literacy-related demands and complexity. Previous studies have highlighted the importance of developing educational programs or strategies to improve individuals' health literacy [28], and there is a need in South Korea for strategies to increase accessibility to health information through the development and provision of effective health information media for adults with IFG. Therefore, strategies considering sex and age are expected to reduce the prevalence of diabetes in individuals with IFG.

In this study, 76.1% of participants with IFG were found to be obese (BMI ≥ 23kg/m^2^). Since there was a very high correlation between an increase in BMI and the prevalence of diabetes mellitus and IFG [5], exercise and healthy eating habits for BMI control are considered important. However, our study found that “walking for 150 minutes or more per week” did not have a significant effect on IFG prevalence. It was also found that about 50% of all participants “walked for 150 minutes or more a week,” indicating that about half of Korean adults have a considerable lack of activity. Studies have reported that the prevalence of IFG increased with a lack of exercise [19, 39]. Therefore, further studies are needed to understand basic metabolism in individuals with IFG, and consider the intensity and degree of exercise that affects the consequences of obesity and BMI.

This study has several limitations. First, since the participants were examined based on responses to a multidimensional questionnaire at a single time point, this study could not analyze in detail the causal relationship between health literacy and health behavior factors affecting IFG. Second, we evaluated health literacy using two items (i.e., “recognition of nutrition fact labels” and “utilization of the nutrition facts labels”). Since an exact measurement of health literacy levels was not used, the relationship between health literacy level and health behavior in those with IFG could not be closely evaluated. Further studies using more detailed surveys are suggested to verify the results of this study.

Despite these potential limitations, our study has several strengths. This study used national statistical data that covered three years, and the participants represented the total adult population in South Korea. The survey design included multi-level sampling, stratification, and clustering. Therefore, the results of this study can be generalized to the adult population of South Korea. In addition, the results confirmed the differences in health behavior and health literacy in the sex of Korean individuals with IFG and those with non-IFG. Particularly, male with IFG have a poor tendency to use health literacy to ensure the practice of health behaviors, such as engaging in exercise, avoiding alcohol, managing obesity, and utilizing food information in this study. Because health literacy is related to health status [40], national policies and support should be focused on mitigating the impacts of low health literacy in male with IFG. Above all, it is important for male with IFG to be aware of the importance of health literacy, so that they can practice effective health behaviors.

## Conclusion

This study found that the prevalence of IFG and factors associated with IFG differed by sex. The predictors of IFG in male were old age, shift work, drinking behavior, frequently dining out, and lower health literacy, while low economic status and obese-level BMI were the IFG predictors in female. To promote health behaviors in individual with IFG, tailored strategies should provide educational interventions for improving health literacy to those with IFG.

## Data Availability

Korea National Health and Nutrition Examination Survey data are available at (https://knhanes.cdc.go.kr/knhanes/main.do) publicly. Entire data from KNHANES are coded and available freely.

## Abbreviations

KNHANES: Korea National Health and Nutrition Examination Survey
KCDC: Korea Centers for Disease Control and Prevention
IFG: Impaired fasting glycemia
